# Role of redox centre in charge transport investigated by novel self-assembled conjugated polymer molecular junctions

**DOI:** 10.1038/ncomms8478

**Published:** 2015-06-18

**Authors:** Zongrui Wang, Huanli Dong, Tao Li, Rune Hviid, Ye Zou, Zhongming Wei, Xiaolong Fu, Erjing Wang, Yonggang Zhen, Kasper Nørgaard, Bo W. Laursen, Wenping Hu

**Affiliations:** 1 Key Laboratory of Organic Solids, Institute of Chemistry, Chinese Academy of Sciences, Beijing 100190, China; 2 Department of Chemistry, Capital Normal University, Beijing 100048, China; 3 Nano-Science Center, Department of Chemistry, University of Copenhagen, Universitetsparken 5, Copenhagen 2100, Denmark; 4 Collaborative Innovation Center of Chemical Science and Engineering, Tianjin University, Tianjin 300072, China

## Abstract

Molecular electronics describes a field that seeks to implement electronic components made of molecular building blocks. To date, few studies have used conjugated polymers in molecular junctions despite the fact that they potentially transport charge more efficiently than the extensively investigated small-molecular systems. Here we report a novel type of molecular tunnelling junction exploring the use of conjugated polymers, which are self-assembled into ultrathin films in a distinguishable ‘planar' manner from the traditional vertically oriented small-molecule monolayers. Electrical measurements on the junctions reveal molecular-specific characteristics of the polymeric molecules in comparison with less conjugated small molecules. More significantly, we decorate redox-active functionality into polymeric backbones, demonstrating a key role of redox centre in the modulation of charge transport behaviour via energy level engineering and external stimuli, and implying the potential of employing tailor-made polymeric components as alternatives to small molecules for future molecular-scale electronics.

## Abstract (web summary)

To date, conjugated polymers have not yet been used in molecular junctions despite the fact that they transport charge more efficiently than small-molecular systems. Here, Wang *et al.* showcase the self-assembly of conjugated polymers into ultrathin films in a planar manner for device construction.

## Graphical abstract

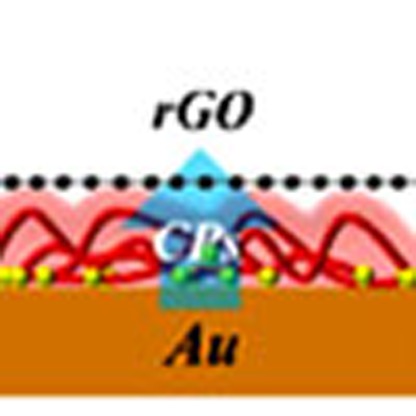

The visionary idea of molecular electronics has been sketched out for decades^1^. Much effort has been devoted to the fabrication of solid-state molecular electronic devices, contributing to great breakthroughs in this multidisciplinary field^2^,^3^,^4^. From an experimental perspective, making contacts to molecular components for device fabrication presents significant challenges^5^,^6^,^7^,^8^,^9^. For this reason, devices based on self-assembled molecular films are more robust for reproducible studies over single-molecule junctions, and they are more likely to be compatible with future mass production and integration^10^,^11^,^12^. On the other hand, from a molecular perspective, π-conjugated molecules have much narrower highest occupied molecular orbital (HOMO)–lowest unoccupied molecular orbital (LUMO) energy gaps in comparison with alkane chains (a benchmark system in the field of molecular electronics) and can transport charge more efficiently^2^,^13^,^14^,^15^. Therefore, conjugated molecules are better candidates for circuit elements, often termed ‘molecular wires'. To date, both individual and self-assembled monolayers (SAMs) of conjugated small molecules have been extensively studied as the active components in tunnelling junctions^16^,^17^. Compared with small molecules, tailor-made π-conjugated polymers (CPs) possess even more extended conjugation length for potentially higher charge transport capabilities, broader optical absorption and better processability. Although CPs and their bulk applications in optoelectronics have received significant attention for decades^18^,^19^,^20^, very limited work has been carried out on incorporating CPs into molecular-scale electronics^21^,^22^. Specifically, to the best of our knowledge, self-assembly of CPs and their application in molecular thin-film junctions have not been studied so far.

Herein, we investigate the self-assembly manner of long CPs on metal surface in comparison with typical vertically aligned small molecules, and thereby introduce a novel type of molecular junction employing CPs as the active charge transport layer. Poly(*p*-phenylene ethynylene)s (PPEs)^21^,^22^,^23^,^24^,^25^ are selected as the model CPs and we designed a new kind of CPs by incorporating redox-active tetrathiafulvalene (TTF) units into the polymeric backbones (TTF–PPEs)^26^ (molecular structures shown in Fig. 1a, the synthesis of the polymers see Methods and Supplementary Figs 1 and 2). Cyclic voltammetry (CV) exhibits TTF characteristic peaks for TTF–PPEs, which are not observed for PPEs (Fig. 1b). End-capped anchoring groups allow the polymeric molecules to be chemisorbed onto metal bottom electrodes. Thin, conductive and flexible graphene materials lend soft top contact to the molecular components^27^,^28^,^29^, which is important for constructing molecular junctions with high device yield and operational stability^30^,^31^,^32^. Experimental evidence indicates that although CPs are self-assembled into ultrathin films (2–3 nm) in a distinguishable ‘planar' manner from the vertically oriented SAMs, they can still implement their molecular-specific characteristics in tunnelling junctions in comparison with the less conjugated small molecules. Furthermore, we demonstrate the role of redox centre in the modulation of charge transport behaviour via energy level engineering and external stimuli. This work innovatively proposes the potential of employing CPs as alternatives to typical small-molecule assemblies for device construction in the field of molecular electronics, as well as their tailorability on device performance via synthetic methods for desirable functionalities.

## Results

### Self-assembled ultrathin films of CPs on Au surface

**Figure 1:**
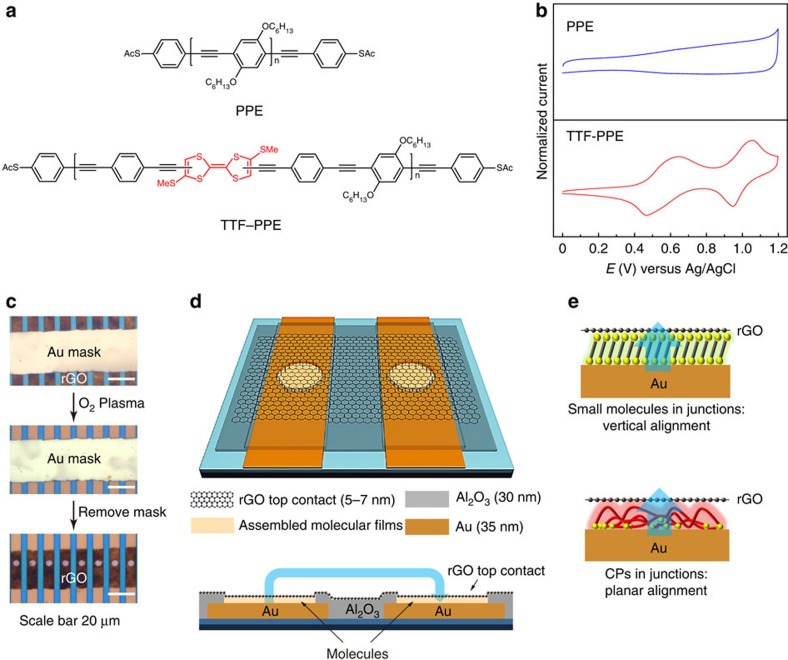
rGO test bed with self-assembled CPs on Au bottom electrodes. (**a**) Chemical structures of PPE and TTF–PPE. (**b**) Cyclic voltammograms of PPEs and TTF–PPEs dissolved in tetrahydrofuran solution. (**c**) Optical images depicting the process of device fabrication using thin Au films as masks to remove the unwanted parts of rGO films. (**d**) Schematic view of rGO top-contact test bed for molecular tunneling junctions. The blue arrow in (**d**) indicates charge transfer between adjacent junctions via graphene top contact. (**e**) Schematic view of self-assembled small molecules and long CPs inside the junctions. The blue arrows in (**e**) indicate charge transfer by direct tunnelling across the molecular barriers.

Both thioacetyl-end-functionalized PPEs (*M*_w_≈51,300, *M*_w_/*M*_n_=3.09) and TTF–PPEs (*M*_w_≈22,800, *M*_w_/*M*_n_=3.1) are chemisorbed on flat Au substrates (root mean squared (r.m.s.) roughness≈0.5 nm) in micropores (4 μm in diameter)^28^. Devices based on SAMs of small molecules (for example, 1-dodecanethiol (C12) and oligo(phenylene ethynylene)s (OPE3)) for control experiments are prepared in the same process. Solution-processed reduced graphene oxide (rGO) films (5–7 nm) are transferred onto the molecular films to form a top contact^28^. Thin Au films (∼100 nm) are utilized as masks by an Au-stripping technique^33^ to remove the unserviceable parts of the rGO films (Fig. 1c). A schematic view of rGO top-contact test bed is shown in Fig. 1d. In this test bed, rGO can perform as both top contacts and conductive interconnects between molecular components. The measurements are carried out in a double-junction geometry without additional top metal electrodes. A schematic view of self-assembled small molecules and long CPs inside the tunnelling junctions is shown in Fig. 1e.

**Figure 2:**
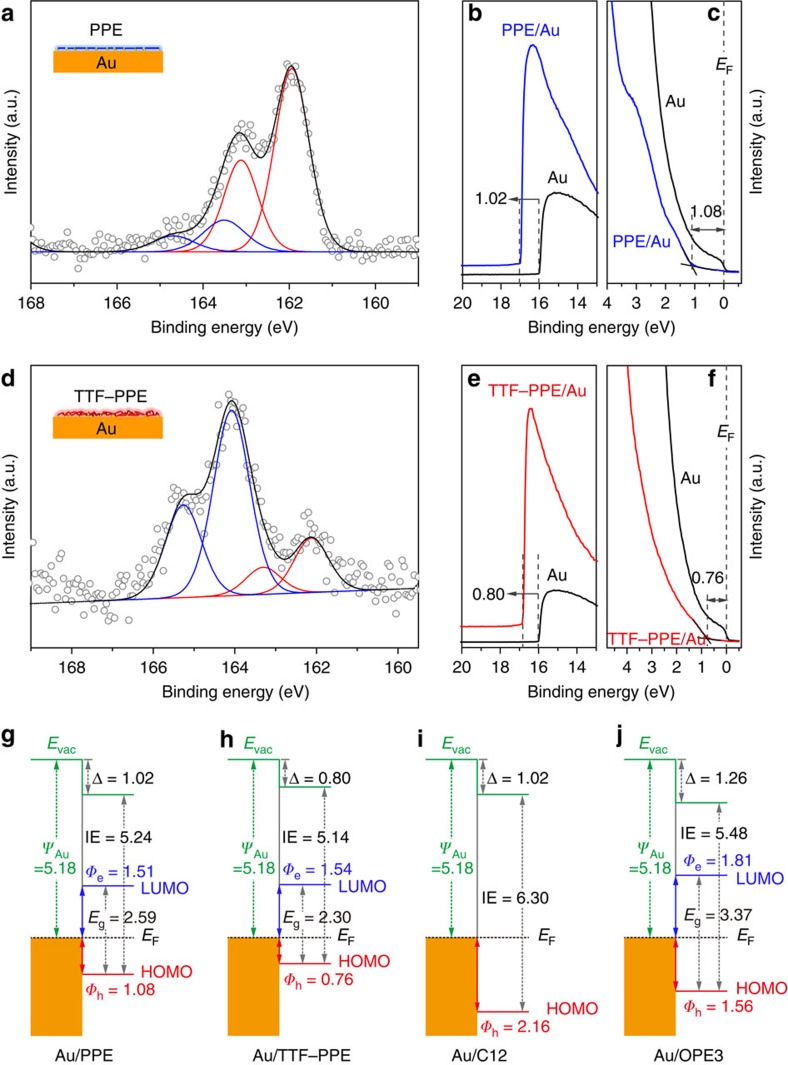
XPS and UPS characterization of self-assembled CP films. S 2*p* XPS spectra for self-assembled films of (**a**) PPEs and (**d**) TTF–PPEs on Au substrate. (**b**,**e**) The measured UPS spectra of the SEC region of PPE/Au and TTF–PPE/Au. (**c**,**f**) The HOMO region of PPE/Au and TTF–PPE/Au. (**g**–**j**) The energy level diagrams of Au/PPE, Au/TTF–PPE, Au/C12 and Au/OPE3, respectively.

Self-assembled films of PPEs and TTF–PPEs were first prepared on large-area Au substrates (1 cm^2^) for ease of characterization. The CP-covered surface morphology was imaged by atomic force microscopy (Supplementary Fig. 3), showing both CP films having r.m.s. roughness below 1 nm. Figure 2a,d depicts X-ray photoelectron spectroscopy (XPS) of S 2*p* spectrum for self-assembled PPE and TTF–PPE films on Au, respectively. For PPEs (S elements only appear in head groups), S 2*p* spectrum showed only one doublet peak with S 2*p*_3/2_ at 162 eV attributed to S atom bonding to Au. Limited signals were observed at 164 eV from S to R (R refers to H/Ac/C), indicating that almost no free anchoring groups were present. For TTF–PPEs, S 2*p* signals were clearly distinguished. In addition to the peaks at 162 eV related to S–Au bonds, much stronger peaks at 164 eV appeared, attributed to S–R bonds in the TTF unit^34^. The film thickness of PPE and TTF–PPE can be calculated to be 2.0±0.2 nm and 3.1±0.2 nm, respectively, based on the attenuation of the Au 4*f* signal (Supplementary Fig. 4). XPS results depict two important following features of the CP films: (i) anchoring groups on both ends of the polymers managed to bond to the Au surface; and (ii) the films thickness is much smaller than the length of the CP backbones (up to several tens of nanometres). Furthermore, scanning tunnelling microscopy images clearly showed that CPs were lying flat on Au surface forming a continuous film (Supplementary Fig. 5). Consequently, we can conclude that long CPs are self-assembled in a ‘planar' manner on metal surface rather than a typical vertical-alignment way for small molecules. Mechanisms and kinetics of SAM formation have been extensively studied^35^. In general, the adsorbed molecules initially form either a disordered mass or an ordered two-dimensional ‘lying down phase' on the substrate surface, and over a period of time, begin to form ordered structures via assembly of both head groups and tail groups^36^. Different from small molecules, it would be much more difficult for long CPs to spontaneously stand up on the substrate surface in the assembly process. Instead, CPs would keep the initial two-dimensional ‘lying down phase', finally organizing into films with comparable thickness to typical small-molecule SAMs. It is noteworthy that the incorporation of TTF units into PPEs decreases the rigidity of the polymeric backbone due to *cis*–*trans* isomerization, thereby increasing the film thickness.

Ultraviolet photoelectron spectroscopy (UPS) was utilized to analyse the band spectra of molecular film/Au interface, especially the barrier height for charge injection, which plays a key role for charge transport in molecular tunnelling junctions. The measured UPS spectra of secondary electron cutoff (SEC) region and the HOMO region for PPEs and TTF–PPEs are shown in Fig. 2b,c and Fig. 2e,f, respectively (UPS spectra of other molecules see Supplementary Fig. 6). Combined with CV and ultraviolet–visible (ultraviolet–vis) absorption spectra, the energy level diagrams of Au/PPE, Au/TTF–PPE, Au/C12 and Au/OPE3 are presented in Fig. 2g–j. Several important features can be clearly identified as following: (i) alkyl chain C12 has fairly large energy gap and injection barrier compared with conjugated systems (OPEs and CPs); (ii) with an increase in conjugation length, the HOMO–LUMO gap decreases within a given molecular series (PPEs/TTF–PPEs versus OPE3); (iii) the incorporation of TTF units into PPE backbone not only further decreases the energy gap, but also realizes a better alignment of energy levels to Au for ease of charge injection (the barrier height for hole injection decreases from 1.08 eV for PPEs to 0.76 eV for TTF–PPEs).

### Electronic characteristics of molecular tunnelling junctions

**Figure 3:**
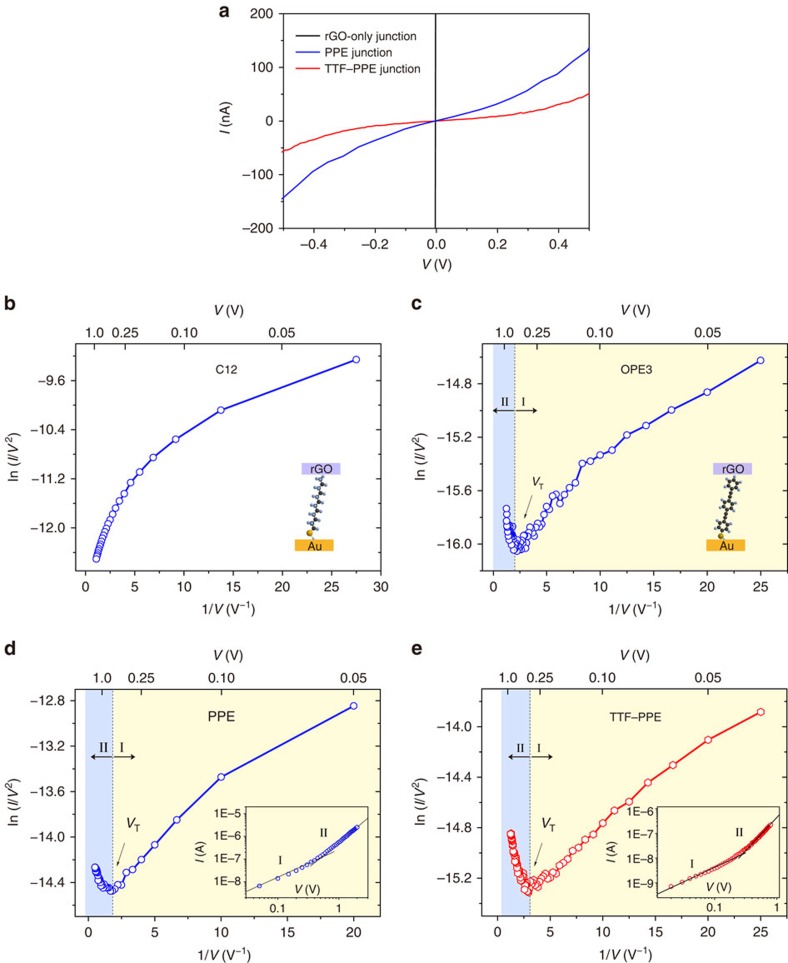
*I*–*V* characteristics of molecular tunnelling junctions. (**a**) Representative *I*–*V* curves of rGO-only junctions and junctions based on PPE and TTF–PPE ultrathin films. (**b**–**e**) Fowler–Nordheim plots for *I*–*V* traces of junctions based on (**b**) C12, (**c**) OPE3, (**d**) PPE and (**e**) TTF–PPE. Two distinct regimes (I and II) are clearly observable in **c**, **d** and **e** with a minimum at *V*_trans_. Insets of **d** and **e** show log–log plot of representative *I*–*V* traces for PPE and TTF–PPE junctions.

On the basis of energy level analysis, both CPs and small molecules were assembled into rGO top-contact test bed for charge transport measurements. rGO-only junctions showed typical ohmic behaviour with resistances orders of magnitude lower than the junctions based on molecular components (Fig. 3a). Typical *I*–*V* curves of the CP junctions are non-linear showing semiconducting characteristics, similar to typical SAM junctions. The junctions were very stable and they did not exhibit either current attenuation or short-circuit failure on repeated bias scans (Supplementary Fig. 7), indicating the superiority of the rGO test bed (shelf-life as long as months) as well as the stability of the polymeric ultrathin films. There is no intuitive difference between *I–V* curves based on PPEs and TTF–PPEs, only that on average the measured tunnelling current of PPE junctions is slightly larger (by a factor of 2–3) than TTF–PPE junctions, resulting from a joint action of tunnelling length and energy barriers (more discussion in the following sections).

As discussed above, the CPs formed a novel type of molecular layer with thickness of 2–3 nm between bottom and top contacts. On this occasion, charge transport in the junctions can be simplified as electron tunnelling through a barrier^37^. In low-bias regions, the tunnelling current can be expressed by


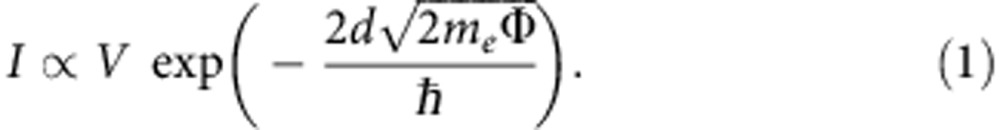


Wherein *d* is the barrier width, *m*_e_ is the electron effective mass and Φ is the barrier height (energy offset between the Fermi level of electrode and the frontier molecular orbital, normally HOMO). A Fowler–Nordheim (F–N) plot of ln (*I*/*V*^2^) versus 1/*V* can provide further insight into the charge transport process—a method called transition voltage spectroscopy^14^,^37^,^38^,^39^,^40^,^41^,^42^. A minimum in the F–N plots corresponds to a crossover between a low- and a high-bias regime at a transition voltage, *V*_trans_. The transition voltage is related to the energy offset between the electrode Fermi level and the nearest molecular orbital (for example, the HOMO). In particular, Bâldea derived an approximate relation, for a point-like molecule, between *V*_trans_ and the energy level offset Φ: *eV*_trans_≈1.15Φ (refs 40, 41). It is generally accepted that transition voltage spectroscopy, combined with UPS analysis, is a reliable method to study the energy level relationship between the electrode Fermi level and closest molecular orbital. Figure 3b–e shows the F–N plots for *I*–*V* curves of junctions based on C12, OPE3, PPEs and TTF–PPEs, respectively. No transition voltage is observed for the C12 control system within the applied bias window (−1 to 1 V), which agrees with the fact that alkanes have fairly large energy gaps (up to 7 eV) and, therefore, large charge injection barriers^43^. While for OPE3, PPE and TTF–PPE junctions, two distinct regimes (I and II) are clearly observed with minima at *V*_trans_. Log–log plot showed consistent results (Fig. 3d,e, inset). *V*_trans_ for OPE3 and PPEs is 0.55±0.1 V and 0.6±0.1 V, respectively, and for TTF–PPEs it is evidently smaller (0.28±0.08 V). The magnitude of *V*_trans_ scales roughly with the *E*_F_–*E*_HOMO_ energy difference predicted by UPS and corresponds to molecular-specific signatures. The observed lowering of the effective barrier height in transition voltage analysis compared with UPS measured values has been discussed elsewhere^14^,^37^,^41^. The agreement between electrical measurement and UPS data further confirms that incorporation of TTF units into PPE backbones realized efficient modulation of charge transport behaviour via altering conjugation path and energy level alignment.

### Modulation of charge transport properties via redox process

In addition to shifting the molecular energy levels, TTF units can act as redox-active centres, and we further investigated how the redox process affects charge transport properties of the junctions. CV characterization in solution and thin-film state both verified that the redox properties were retained after the TTF units were inserted into PPE backbones (Fig. 1b; Supplementary Fig. 8). On this basis, both chemical and electrochemical methods were employed to induce redox changes within CPs.

**Figure 4:**
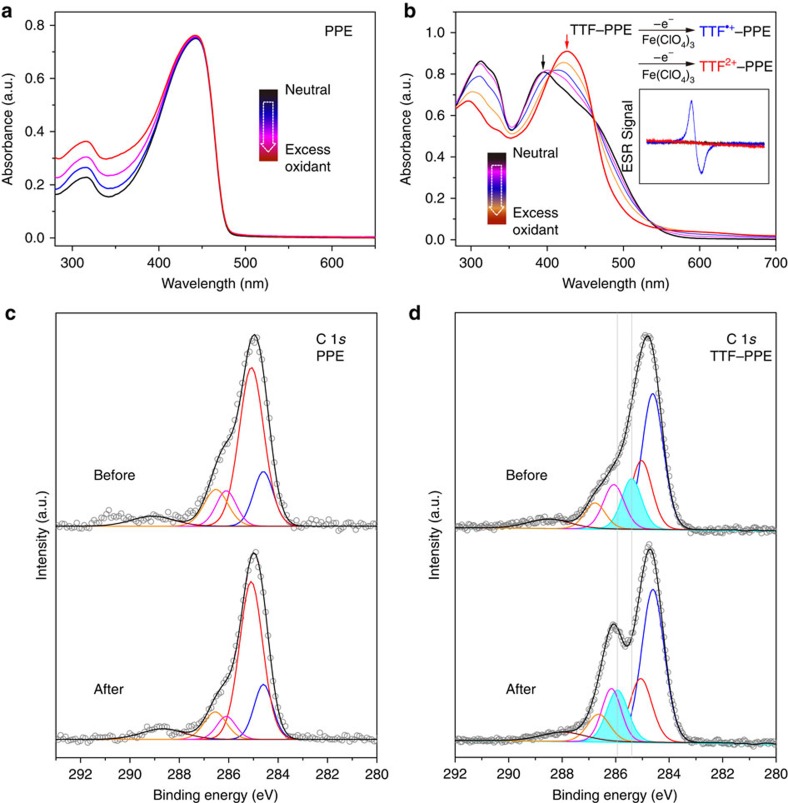
Characterization of redox changes within TTF–PPEs. Ultraviolet–vis absorption spectra changes (in anhydrous THF, 298 K) on successively adding oxidant Fe(ClO_4_)_3_·6H_2_O in solutions of (**a**) PPE and (**b**) TTF–PPE. The initial state TTF–PPE (black) and final dication state TTF^2+^–PPE (red) are indicated in bold. (**b**) Inset, the change of ESR spectrum with the addition of oxidant and blue curve refers to the radical state TTF^·+^–PPE. (**c**) and (**d**) C 1*s* XPS spectra for self-assembled films of PPE and TTF–PPE before and after oxidant treatment, respectively. A clear change in C 1*s* spectra for TTF–PPE films can be observed, which is absent for that of PPE.

When adding oxidizing agent iron perchlorate hexahydrate Fe(ClO_4_)_3_·6H_2_O in CP solutions, ultraviolet–vis spectroscopy showed clearly changed absorption spectrum of TTF–PPEs, while that of the PPEs was not affected (Fig. 4a,b). TTF is a nonaromatic 14e structure, which readily loses two electrons to form a stable aromatic structure. The oxidition of TTF–PPE approached saturation when excess Fe(ClO_4_)_3_·6H_2_O was used, ascribed to TTF^2+^–PPE. The ultraviolet–vis spectroscopy did not exhibit a stable radical cation state^44^,^45^,^46^ (TTF^·+^–PPE), probably due to the poor stability of the radical cations at our experimental conditions, especially in the dilute solution (10^−5^ M) suitable for ultraviolet–vis measurement. The radical cations were successfully traced by electron spin-resonance spectroscopy (ESR) analysis with a distinct signal appearing during the addition of oxidant^47^,^48^ (Fig. 4b; inset), whose half-life period was estimated to be 53 min in the 10^−3^ M tetrahydrofuran (THF) solution (Supplementary Fig. 9), indicating their short lifetime and instability. As for CP films on Au substrate, we analysed the C 1*s* XPS spectra of PPEs (Fig. 4c) and TTF–PPEs (Fig. 4d) before and after oxidant treatment (the corresponding S 2*p* spectra see Supplementary Fig. 10). After immersing in Fe(ClO_4_)_3_ solution for 12 h, no visible change on C 1*s* spectra was observed for PPEs. At the same time, an obvious change of C 1*s* signal for TTF–PPEs appeared after oxidant treatment: besides the main peak in the spectrum falling at 284.6 eV attributed to unsaturated carbons belonging to the polymer backbone, which resembles that of PPEs, a pronounced shoulder peak at around 286.1 eV appeared. This phenomenon was induced by an upper shift of C–S bonding energy from 285.3 eV (ref. 49) to 285.9 eV after the TTF unit loses two electrons. The C–S peak (shown in green) with ∼0.6 eV shift is depicted in Fig. 4d. No detectable signal from Fe was observed, excluding any side effects from the oxidant on the XPS results.

**Figure 5:**
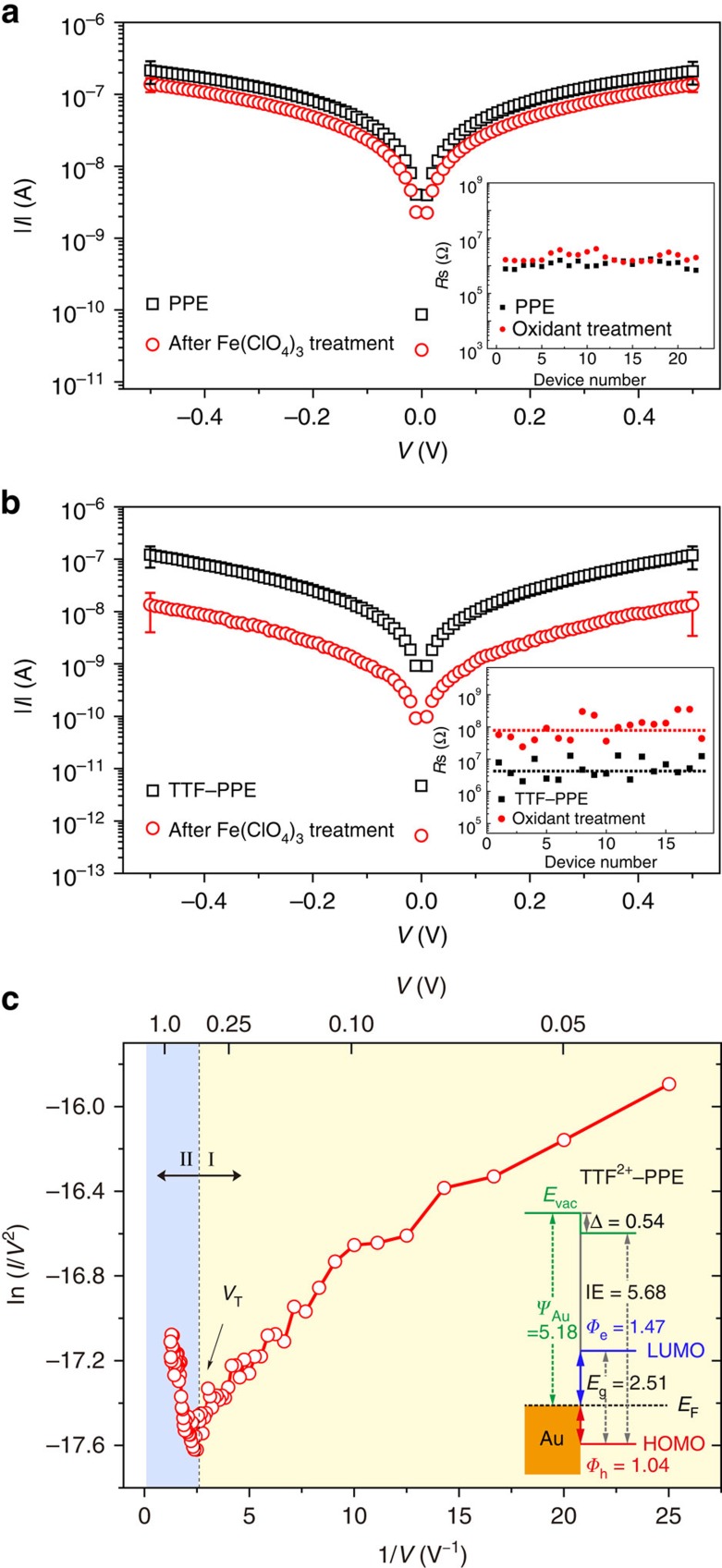
Modulation of TTF–PPE junctions via chemical oxidation. *I–V* characteristics of (**a**) PPE and (**b**) TTF–PPE junctions before/as-prepared (black square) and after treated with excess iron perchlorate hexahydrate (red circle). The plots are generated from average values obtained from least 20 junctions of the same batch. Insets of **a** and **b** show statistics on sheet resistance of the PPE and TTF–PPE junctions, respectively. (**c**) Fowler–Nordheim plot for *I*–*V* traces of TTF^2+^–PPE junctions. Inset shows the energy level diagram of TTF^2+^–PPE/Au.

The electronic properties of CP films before and after chemical oxidation were measured using the above-mentioned rGO top-contact test bed. The sheet resistance of PPE and TTF–PPE junctions (inset of Fig. 5a,b) is calculated from low-bias regimes (−0.1 V≤*V*≤0.1 V) of *I*(*V*) characteristics. Experimental evidences confirmed that a modulation of conductance of TTF–PPE junctions was achieved via oxidant treatment on the CP films, which was not observed for non-redox-active PPE junctions. The *R*s of TTF^2+^–PPE junction was on average one order of magnitude higher than that of their neutral state (Fig. 5b). F–N plots indicated *V*_trans_ at 0.37±0.08 V (Fig. 5c), ∼0.1 V larger than that observed before oxidation. UPS revealed a much larger barrier for hole injection after the oxidant treatment (up to 1.04 eV compared with 0.76 eV, Fig. 5c; inset). In addition, from the absorption spectra (Fig. 4b), the energy gap was enlarged via oxidation (from 2.30 to 2.51 eV). No characteristic changes on conductance of PPE junctions were recorded on oxidant treatment, confirming that the modulating effect is originated from the redox changes of TTF unit. The active role of redox centre in the modulation of device performance also further confirms the contribution from polymeric backbones dominates the charge transport process, agreeing well with previous discussions. A summary of energy levels and *V*_trans_ for CPs and small molecules in this work is shown in Table 1.

Although CV characterizations verified a reversible redox process for TTF–PPEs both in solution and thin-film form, it turned out to be difficult to reduce TTF^2+^–PPE back to the neutral state using solely chemical reducing agent. As discussed above, the dicationic state has aromatic structures with higher stability. Therefore, the reduction process is very likely less preferred. Ferrocene has been used as reductant for small-molecule TTF compounds in solution^46^, but was not applicable for CPs in our experiment (Supplementary Fig. 11). Other reducing agents such as ascorbic acid was also tried without success (insoluble in THF).

**Figure 6:**
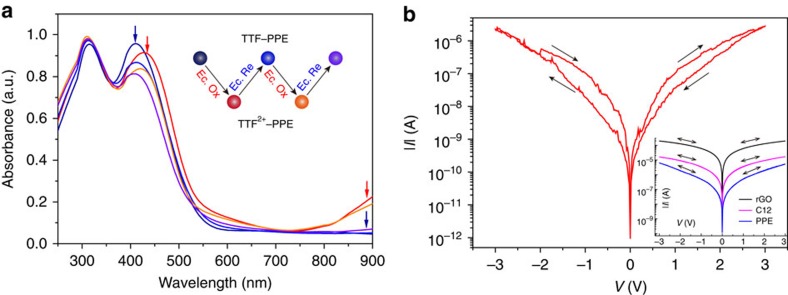
Modulation of TTF–PPE junctions via electrochemical method. (**a**) Ultraviolet–vis absorption spectra (in anhydrous THF, 0.1 M Bu_4_NPF_6_, 298 K) of TTF–PPEs (blue), after electrochemical oxidation at +2 V (versus Ag/AgCl) for 10 min (red) and subsequent electrochemical reduction at −0.2 V (versus Ag/AgCl) for 30 min (cyan). A cycling of oxidation–reduction process is achieved. Repeated electrochemical oxidation and reduction process is realized in the same way: at +2 V (versus Ag/AgCl) for 10 min (yellow) for oxidation and at −0.2 V (versus Ag/AgCl) for 30 min (violet) for reduction, respectively. (**b**) Typical *I–V* characteristics of TTF–PPE junction under larger applied bias sweeps. Hysteretic conductance switching following an ‘oxidation-current decrease-reduction-current recovery' trend indicates an oxidation–reduction process within the device. Inset, no hysteresis loops were observed in control experiments with rGO-only, C12 and PPE junctions.

On this occasion, electrochemical method was employed for a reversible oxidation–reduction process. As shown in Fig. 6a, the cycling process was successfully achieved in solution by recording, in real-time, ultraviolet–vis absorption spectra of TTF–PPEs on electrochemical oxidation–reduction process in a spectroelectrochemical cell (Supplementary Fig. 12). With CVs as a reference, TTF–PPEs can be oxidized into TTF^2+^–PPEs by applying a potential at +2.0 V (versus Ag/AgCl) for 10 min, where a new absorption peak at about 436 nm and an broad absorption around at 1,050 nm (Supplementary Fig. 13) appeared. This peak shift agreed with that recorded by chemical oxidation method (Fig. 4b), only with a few nanometres displacement probably due to the influence of the electrolyte environment. By sequential application of a reduction potential at −0.2 V (versus Ag/AgCl) for 30 min, the spectrum changed back to the original TTF–PPEs state, indicating the reduction of TTF^2+^–PPEs to the neutral TTF–PPEs. The electrochemical oxidation and reduction process can be repeated for cycles (Supplementary Fig. 13).

As for solid-state molecular junctions, redox changes can be induced by applying larger bias voltages^31^,^50^, analogous to the oxidation/reduction potentials supplied by the electrochemical workstation. Due to a special geometry of the rGO top-contact test bed, no metal deposition was made on top of the junction. Therefore, there is no need to worry about the formation of metal filaments during device fabrication and operation, even when a relatively large bias is applied. Generally, the device can be safely run in the −5 to 5 V window without failure. Figure 6b shows hysteretic conductance switching characteristics of TTF–PPE junction under larger applied bias sweeps (>2 V), which was not observed in control devices based on rGO only, C12 and PPEs (Fig. 6b; inset). The hysteresis loops generally followed an ‘oxidation-current decrease-reduction-current recovery' trend. It agreed well with the previous discussions and thus confirmed the reversibility of redox process in TTF–PPE devices. WRER (write-read-erase-read) operations were also attempted by pulsing +4 and −4 V for 30 s each time and reading at +1 V, although the operation stability is poorer than the small-molecule devices (Supplementary Fig. 14).

## Discussion

Different from previous work focusing on monolayered small-molecule devices, we introduce a new type of thin-film tunnelling junction based on self-assembled CPs. Although long CPs are aligned in a different way from small molecules when they are chemisorbed on metal surface, it is interesting to see that they can organize into films with comparable thickness to that of typical SAMs, and this novel type of planar layer possesses molecular-specific characteristics (band gap, conjugation path and so on) that can be ‘read' by electrical measurements. Furthermore, we demonstrate the potential of tailoring the charge transport behaviours of the polymeric components by synthetic methods. The synthetic tailorability of functional molecules not only enables energy level engineering when they are assembled between contact electrodes, but also modulation of their chemical/physical properties via external stimuli. Our work could lead to more studies and potential applications on incorporating CPs into molecular-scale devices for electrical switches and memories, preferably taking advantages of unique properties of CPs (for example, larger charge transport capabilities and better processability) for a potential alternative to SAMs of small molecules.

Although the electron transport through planar CPs might not be as simple as we proposed, it is generally accepted that in this short range (<3 nm), non-resonant tunnelling would dominate. In this sense, the electron has no real retention time on the CPs (for example, hopping into the empty states created by oxidation) and the junction conductance will be determined only by the height of tunnelling barrier and tunnelling distance (thickness of the CP film). For further confirmation, we synthesized a group of ‘shorter' PPEs (*n*∼15 and *n*∼10) for control experiments to investigate whether there is lateral propagation of electrons within the film. We suppose that if these shorter PPEs are also lying flat on the Au surface, we can, thus, compare their charge transport properties with long PPEs. If the short PPEs exhibit apparently different characteristics compared with the long PPEs, a lateral charge propagation may be involved. However, based on XPS analysis, these shorter PPEs were found to be more inclined to stand up rather than totally lie flat on the Au surface (Supplementary Fig. 15), thus exhibiting lower conductance comparing with the aforementioned PPEs (*n*∼100) (Supplementary Fig. 16). It would be interesting to study at what polymerization degree that the polymer wires would begin to lie totally flat on the surface. Further work is already on the way.

Another point worthy of attention is that the electrochemical method appears to be superior to the chemical method for tuning the performance of TTF–PPE junctions. On one hand, cycling oxidation–reduction process can be much easier achieved using an electrochemical method; on the other hand, the encapsulated geometry of the molecular junctions is designed under the consideration of long shelf-life, stable operation and possible scalability. Using chemicals would not be the best choice for the operation of solid-state devices. At the present state, the device performance under electrical oxidation/reduction is not as stable as the reported small-molecule systems, which indicates there is still room for improvement on molecular design and calls for a deeper understanding of specific molecular charateristics for a better device manipulation. More importantly, in addition to redox-active systems, polymeric molecules endowed with more complex functionalities and improved self-assembly ability can be expected for constructing nanoscale electronic switchable devices on various external stimuli.

In summary, we study the self-assembly of two kinds of CPs: PPEs and redox-active TTF–PPEs, on Au surface. The long CPs are self-assembled into ultrathin films (2–3 nm) in a distinguishable ‘planar' manner from the vertically oriented SAMs. On this basis, we introduce a novel type of CP junction that exhibits molecular-specific properties of the CPs on electrical measurements in comparison with conventional small molecules. More significantly, it is confirmed that the decoration of TTF units into the PPE backbone not only decreases its energy gap but also achieves a better alignment of energy levels for charge injection. Furthermore, we demonstrate a key role of the TTF redox centre in the modulation of charge transport through CP junctions using both chemical and electrochemical methods, which is not observed for non-redox-active molecular systems. This work proposes the potential of employing CPs as alternatives to typical small-molecule monolayers for device construction in the field of molecular electronics, as well as the possibility of incorporating desirable functionality into polymeric molecules for switchable electronic devices, which are regarded as the basic elements in the blueprint of molecular electronics.

## Methods

### Materials and characterization

Thioacetyl-end-functionalized PPEs were synthesized according to previously reported methods^23^,^25^ (Supplementary Fig. 1). Thioacetyl-end-functionalized PPEs were synthesized by the polymerization of 1,4-bis(hexyloxy)-2,5-diiodobenzene (M1) and 1,4-diethynyl-2,5-bis(hexyloxy)benzene (M2) monomers through Sonogashira Pd–Cu Coupling reaction (Supplementary Fig. 1). In a 100-ml flask, M2 monomer (0.0606, g, 0.18 mmol), Pd(PPh_3_)_2_Cl_2_ (10.7 mg, 9.2 × 10^−3^ mmol), CuI (2 mg, 0.01 mmol) and end-capping reagent of *S*-4-iodophenyl ethanethioate (2.6 mg, 9.2 × 10^−3^ mmol) were fully mixed, degassed three times and backfilled with N_2_, and then triethylamine (2 ml) in dry toluene (12 ml) solution was added. After stirring the resulted solution for 30 min at 50 °C, M1 monomer (0.09 g, 0.17 mmol) was added for initiating the cross-coupling reaction and continuously stirring for more 4.5 h. A toluene solution of excessive end-capping reagent of *S*-4-iodophenyl ethanethiolate (34 mg, 0.12 mmol) was dissolved and reacted for 40 h. After removing the solvent under reduced pressure, the residue was extracted by THF, precipitated in acetone and filtrated. After purification for several times using THF and acetone, the final PPE orange solids were obtained with a yield of 90%. ^1^H NMR (400 MHz, CDCl_3_, 25 °C) δ 7.01 (bs), 4.05–4.02 (m), 2.44 (m), 1.89–1.83 (m), 1.54–1.51 (m), 1.35–1.34 (m), 0.91–0.89 (m).

Thioacetyl-end-functionalized TTF–PPEs were synthesized and characterized as reported in a previous paper^26^ (Supplementary Fig. 2). Thioacetyl-end-functionalized TTF–PPEs were synthesized by the polymerization of monomer 1,4-bis(hexyloxy)-2,5-diiodobenzene (M1) and monomer ethynylbenzene-functionalized TTF (M3) through Sonogashira Pd–Cu Coupling reaction (Supplementary Fig. 2). The solution of monomer M1 (0. 146 g, 0.276 mmol), monomer M3 (0.180 g, 0.331 mmol), Pd(PPh_3_)_2_Cl_2_ (18.5 mg, 0.026 mmol) and CuI (5.0 mg, 0.026 mmol) were fully mixed in anhydrous THF (40 ml) under N_2_, and then triethylamine (15 ml) was added. After reaction for about 4 days at 60 °C, appropriate additional catalysts and excess end-capping reagent *S*-4-iodophenyl ethanethiolate (46 mg, 0.165 mmol) were added and the reaction mixture was further stirred for another 2 days. After filtration to get rid of the formed precipitate, a red solid was acquired via removing the solvent under rotary evaporation, which was again redissolved with THF as little as possible. TTF–PPE was obtained through reprecipitation separately in 200 ml acetone and 200 ml MeOH, and dried in vacuum. Yield 214 mg, 95%. ^1^H NMR (400 MHz, CDCl_3_, 25 °C): δ 7.47 (m), 7.01 (s), 4.01 (t), 2.53 (s), 2.43 (s), 1.80–1.87 (m), 1.34–1.36 (m), 0.83–0.92 (t).

Other chemicals and reagents were purchased from commercial suppliers and used without further purification.

Ultraviolet–vis absorption was conducted on a JASCO V-570 ultraviolet–vis spectrometer. Cyclic voltammetry (CHI 660C electrochemistry station) was performed under inert conditions in a standard three-electrode electrochemical cell that consisted of a glassy carbon working electrode, an Ag/AgCl reference electrode and a Pt wire counter electrode. Spectroelectrochemical experiments (with JASCO V-570 ultraviolet–vis spectrometer and CHI 660C electrochemistry station) were performed at room temperature in an optically transparent thin-layer electrochemical cell equipped with Pt gauze working electrode, Pt wire counter electrode and Ag/AgCl reference electrode with the optical path length of 1.0 mm. Absorption spectra were recorded immediately after the completion of electrochemical oxidation/reduction process. ESR was recorded on a Bruker ESP-300 spectrometer at the X-band. ESR samples (∼1 mg ml^−1^ in anhydrous THF) were transferred in a quartz flat cell, with the typical spectrometer settings as modulation amplitude of 0.05–0.15 mT, modulation frequency of 100 kHz, microwave power of 12.9 mW, microwave frequency of 9.5 GHz, conversion time of 82 ms, time constant of 164 ms and receiver gain of 10^4^–10^6^.

Self-assembled molecular thin films were grown on flat Au substrates by Au–thiol attachment chemistry^35^. Briefly, CPs (0.1 mg ml^−1^) and OPE3 (0.4 mg ml^−1^) were dissolved in anhydrous THF solution containing 25% triethylamine (Et_3_N, v/v), and C12 was directly dissolved in ethanol (∼2 mM). The Au substrates were then put into the solutions and stored in N_2_ glove box for 48 h. The samples were then taken out and rinsed with excess solvents. The oxidant treatment was performed by immersing the sample into 10 mM Fe(ClO_4_)_3_ in THF. A solution of 10 mM ferrocene was used for reduction treatment.

XPS (KRATOS Axis Ultra DLD spectrometer) was conducted with a monochromatized Al Kα X-ray source (1486.6 eV) and an analyser pass energy of 20 eV at takeoff angles of 90°. All samples were energy calibrated using the Au 4*f*_7/2_ line at 84.0 eV. UPS measurements were taken with the same system with a base pressure >2 × 10^−9^ torr and He I (h=21.22 eV) as the excitation source. To obtain the SEC, a sample bias of −9 V was applied in the normal emission geometry. For all the UPS spectra, the Fermi level of the system was calibrated by determining the Fermi level edge of a sputtered clean Au film and referenced as the zero-binding energy. Atomic force microscopy characterization was performed on a Nanoscopy IIIa (USA) in a tapping mode.

### Device fabrication and electrical measurements

Devices were fabricated according to the previously reported methods^28^. The bottom electrodes were made by ultraviolet–lithography where 20 nm Ti followed by 20 nm Au was thermally evaporated at very low deposition rates (0.1 Å s^−1^). The sample was then loaded in a Savannah 100 atomic layer deposition system from Cambridge Nano Tech. The sample was heated to 110 °C and exposed to alternating gasses of water and trimethylaluminium this was repeated 400 times resulting in a ∼30-nm thick layer of Al_2_O_3_. The microholes were etched using electron beam lithography followed by a wet etch in an aluminium etching mixture consisting of phosphoric and acetic acid.

Monolayered graphene oxide sheets were obtained by Hummers' method. Continuous GO films on SiO_2_/Si substrates were prepared by spin-coating method, which were thermally reduced to rGO films at 600 °C in Ar atmosphere (140 ml min^−1^) for 60 min. The delamination of rGO films from SiO_2_/Si substrate was achieved by generally used methods. Briefly, sodium hydroxide (NaOH) solution (1 M) was slowly added onto the rGO film until the solution spread over the SiO_2_/Si wafer. Then, the wafer was submerged into deionized water at a tilt angle, which delaminated the rGO films from the substrate. The floating rGO films on water were transferred onto the bottom electrodes covered by molecular films to act as the top contact. Finally, the unserviceable parts of rGO films were etched away by oxygen plasma using a stripped Au film as mask. *I*–*V* characterization was carried out using a Keithley 4,200 SCS system and a Micromanipulator 6150 probe station under ambient conditions.

## Additional information

**How to cite this article:** Wang, Z. *et al.* Role of redox centre in charge transport investigated by novel self-assembled conjugated polymer molecular junctions. *Nat. Commun.* 6:7478 doi: 10.1038/ncomms8478 (2015).

## Supplementary Material

Supplementary Information Supplementary Figures 1-16 and Supplementary References

## Figures and Tables

**Table 1:** Summary of orbital energy levels and transition voltage of PPE, TTF–PPE, TTF^2+^–PPE, C12 and OPE3.

**Entry**	***E***_g_* **(eV)**	**UPS**		**Injection barrier (eV)**		***V***_***t*****rans**_**/V**	**Exp. or Calc.**	
		**HOMO**† **(eV)**	**LUMO**‡ **(eV)**	**Hole**§	**Electron**||		**HOMO (eV)**	**LUMO (eV)**
C12	∼7 (ref. 40)	−6.30	—	2.16	—	—	—	—
OPE3	3.37 (ref. 34)	−5.48	−2.11	1.56	1.81	0.55±0.1	−5.31 (ref. 34)	−1.77 (ref. 34)
PPE	2.59	−5.24	−2.65	1.08	1.51	0.6±0.1	−6.30¶, (ref. 24)	−3.71‡
TTF–PPE	2.30	−5.14	−2.84	0.76	1.54	0.28±0.08	−4.84¶	−2.54‡
TTF^2+^–PPE	2.51	−5.68	−3.17	1.04	1.47	0.37±0.08	—	—

Calc., density functional theory (DFT) calculation; Exp., experiment; HOMO, highest occupied molecular orbital; LUMO, lowest unoccupied molecular orbital; PPE, thioacetyl-end-functionalized poly(*p*-phenylene ethynylene); TTF, tetrathiafulvalene; UPS, ultraviolet photoelectron spectroscopy.

^*^E_g_ refers to the optical energy gap.

^†^*E*_HOMO_ is calculated from the UPS spectra of the self-assembled monolayers or ultrathin films on Au surface.

^‡^*E*_LUMO_=*E*_HOMO_+*E*_g_.

^§^The energy barrier between *E*_HOMO,UPS_ and *E*_F_.

^||^The energy barrier between *E*_LUMO,UPS_ and *E*_F_.

^¶^*E*_HOMO_ is calculated from CVs and defined as −(*eE*^ox^_onset_+4.4 eV). Here *E*^ox^_onset_ represents the onset potential for the oxidation.
