# Supplementary material for: Role of redox centre in charge transport investigated by novel self-assembled conjugated polymer molecular junctions
Source: Nat Commun. 2015 Jun 18;6:7478. doi: 10.1038/ncomms8478 (PMC4557373; doi:10.1038/ncomms8478)
Supplement: Supplementary Information — Supplementary Figures 1-16 and Supplementary References [file ncomms8478-s1.pdf]

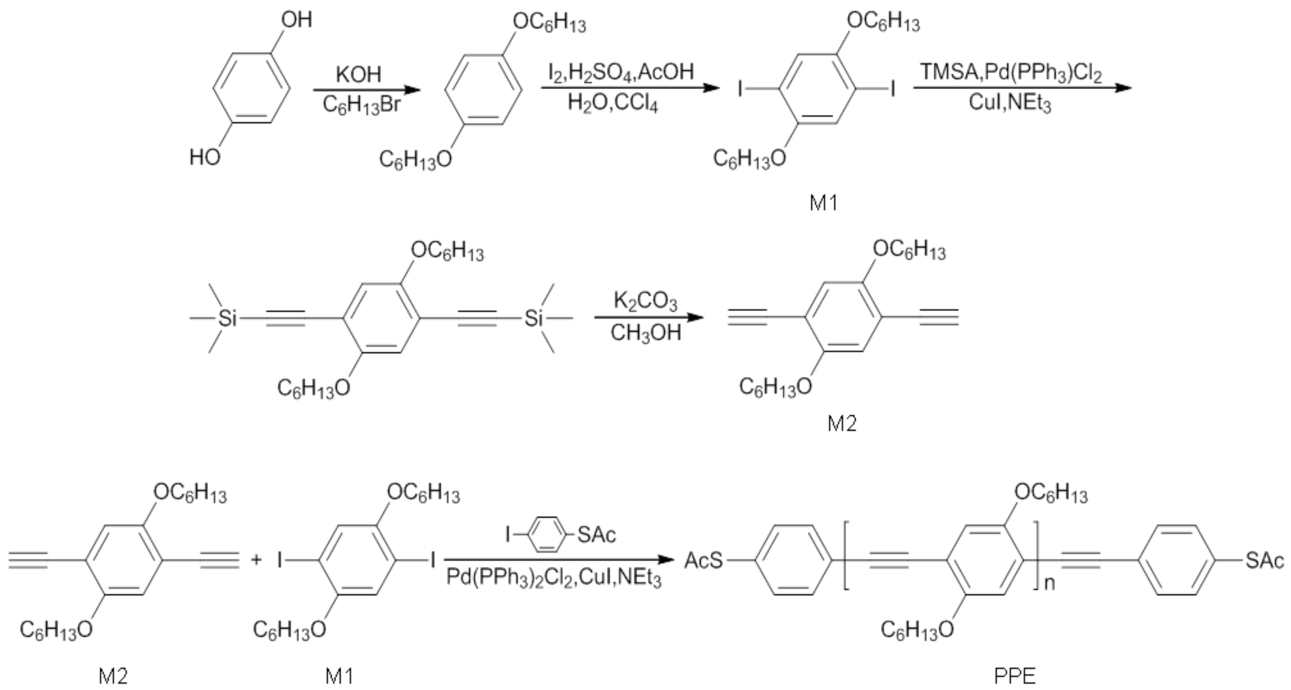

Supplementary Figure 1. A schematic of the synthetic procedure for thioacetyl-end-functionalized PPEs ($M_w \approx 51300$, $M_w/M_n = 3.09$).

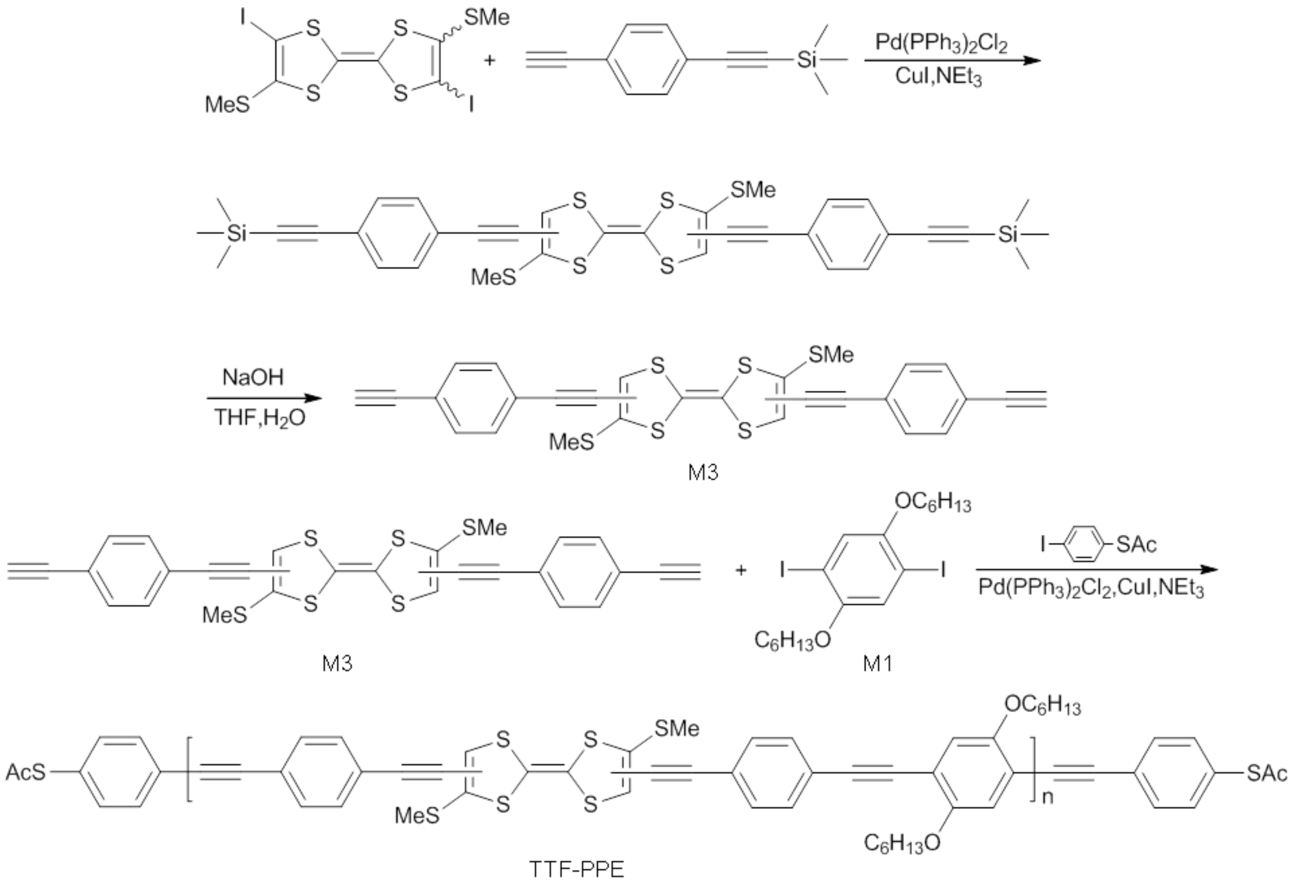

Supplementary Figure 2. A schematic of the synthetic procedure for thioacetyl-end-functionalized TTF-PPEs ($M_w \approx 22800$, $M_w/M_n = 3.1$).

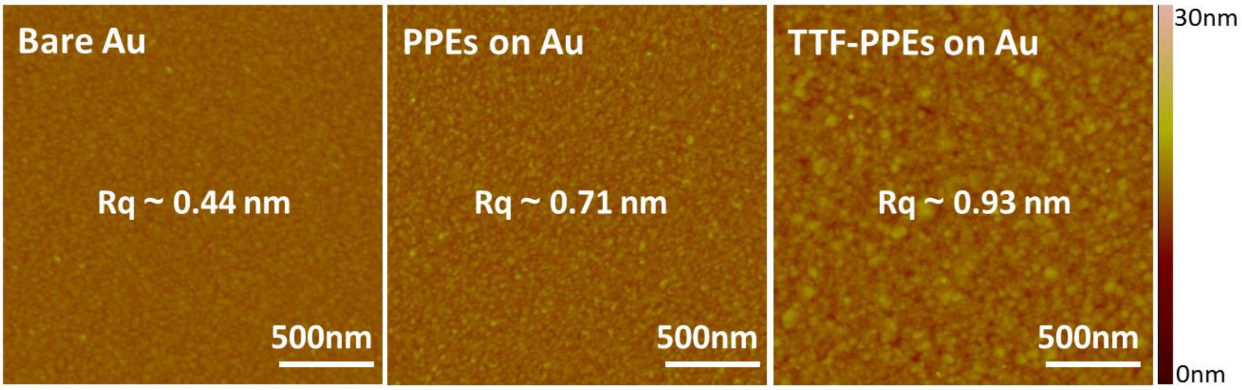

Supplementary Figure 3. AFM images of bare Au surface (left) and with self-assembled films of PPEs (middle), TTF-PPEs (right), respectively.

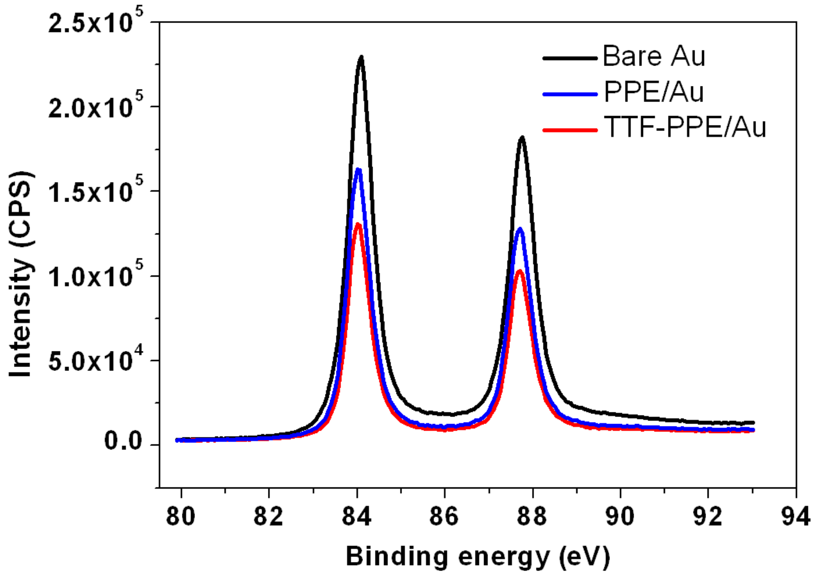

Supplementary Figure 4. XPS spectra of Au 4f region for bare gold and with self-assembled CPs (PPE thickness: 2.0 ± 0.2nm; TTF-PPE thickness: 3.1 ± 0.2nm). The thickness of the film is estimated using the attentuation of the Au 4f signal from the gold surface[1,2] according to the equation (the angle of photoelectron detection was 90°): $d = \lambda \ln(I_{\mathrm{Au}}/I_{\mathrm{SAM}})$ where d is the SAM thickness, λ is the effective attentuation length of photoelectron (4.2 nm) and I_{Au} and I_{SAM} are the average intensities of Au $4f_{5/2}$ and $4f_{7/2}$ signal before and after SAM formation, respectively.

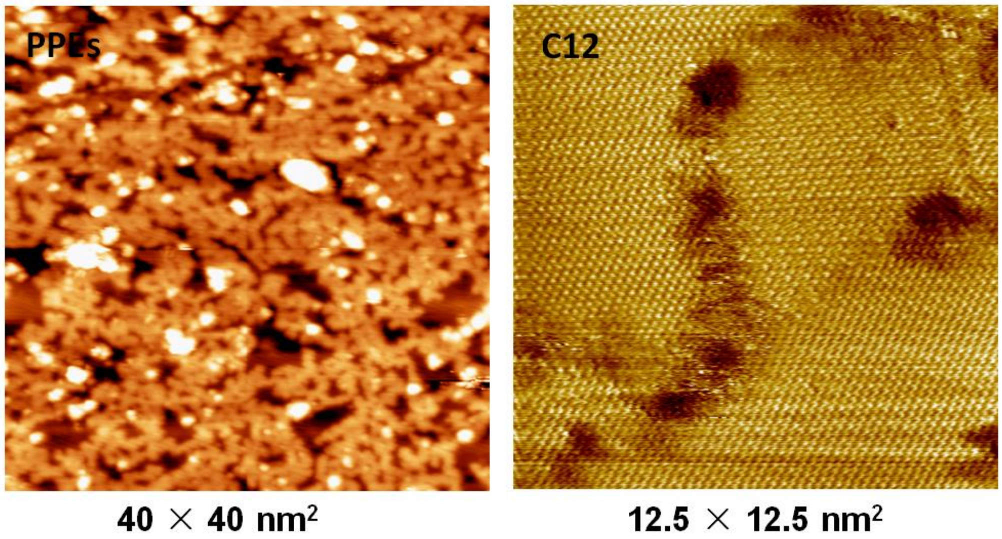

Supplementary Figure 5. STM images of PPE CPs and C12 SAMs on gold surface.

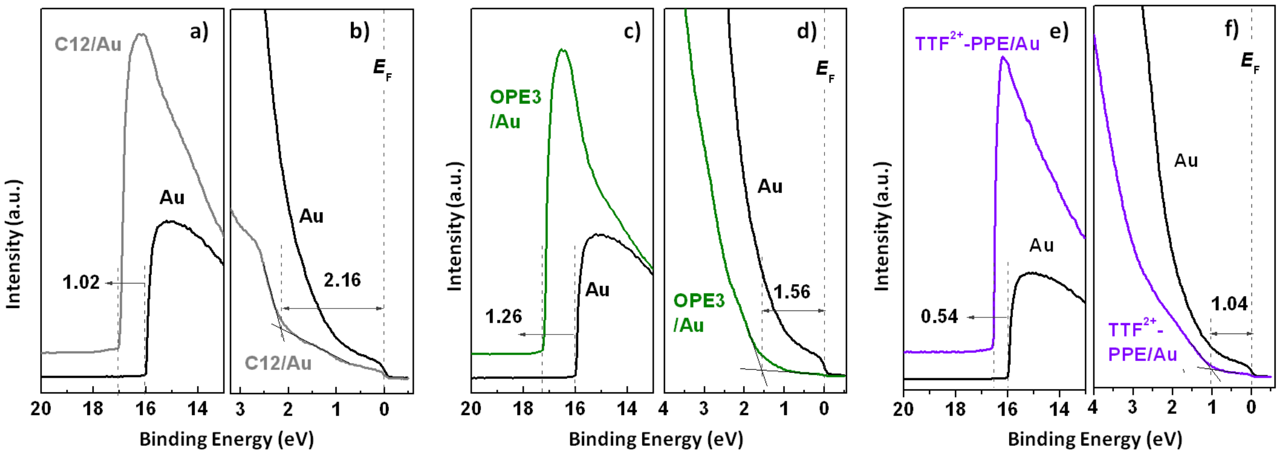

Supplementary Figure 6. The measured UPS spectra of the SEC region of (**a**) Au/C12, (**c**) Au/OPE3, (**e**) Au/TTF^{2+}-PPE, and the HOMO region of (**b**) Au/C12, (**d**) Au/OPE3, (**f**) Au/TTF^{2+}-PPE.

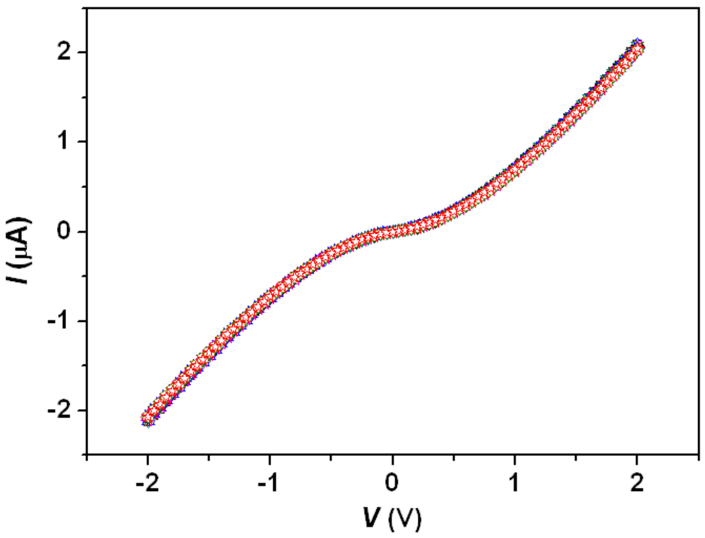

Supplementary Figure 7. Functional stability of the junctions with TTF-PPEs: the junction was tested under potential scans from -2 V to +2 V for 40 times.

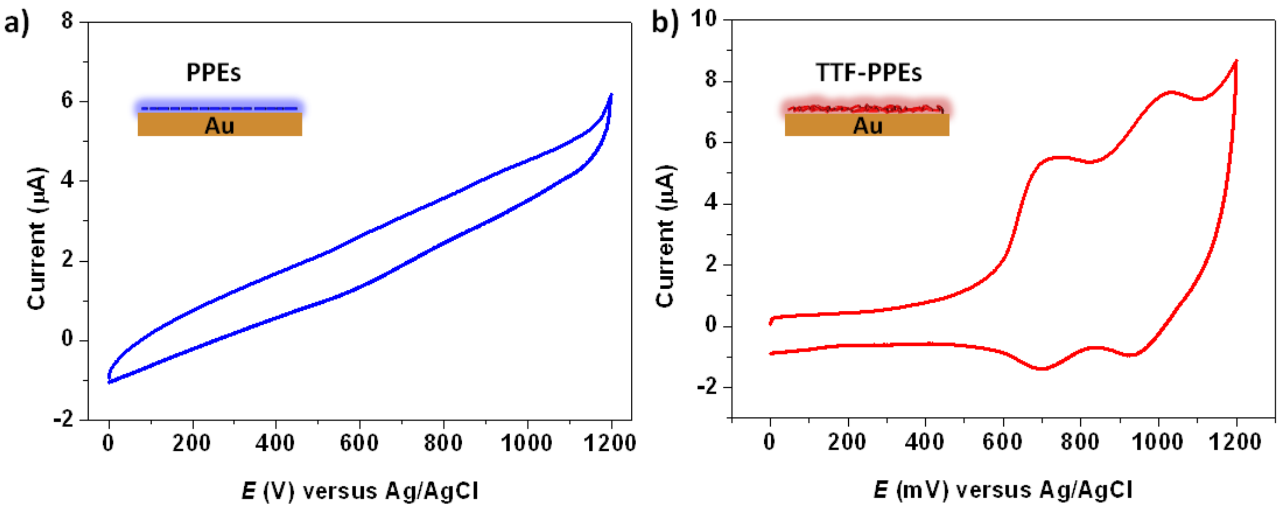

Supplementary Figure 8. Cyclic voltammograms of (**a**) PPEs, (**b**) TTF-PPEs (E^1_{OX} = 681 mV and E^2_{OX} = 1011 mV) on Au surface in THF containing 0.1 M Bu_4NPF_6 as supporting electrolyte at a scan rate of 100 mV/s. After bonding to the Au surface, the oxidation of TTF units would introduce more positive charge and thus increase the system's electrostatic potential energy, resulting that the oxidation process became less energetically favorable (E^1_{OX}=618 mV and E^2_{OX}=1050 mV in solution, see Fig. 1b). On the other hand, it verifies the formation of CP films on Au. The HOMO of TTF-PPE CPs can be estimated as -5.0 eV from the onset potential for the oxidation.

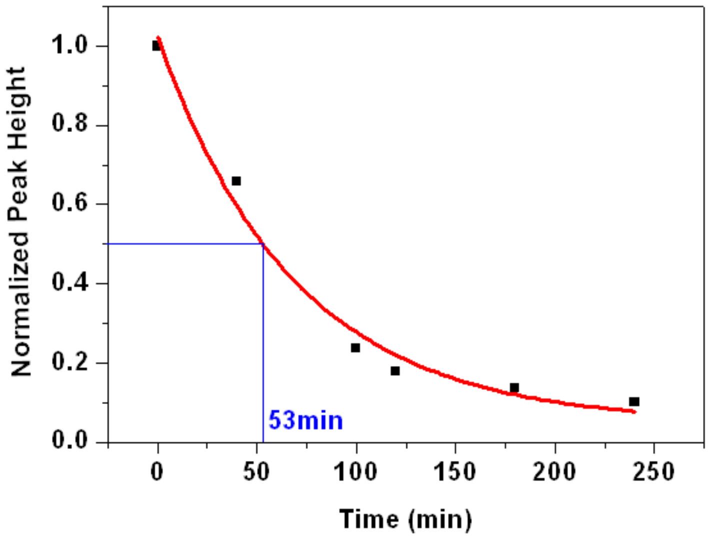

Supplementary Figure 9. The attenuation of free radical $TTF^{\cdot +}$-PPE signal with the time and its half-life time is estimated to be 53 min.

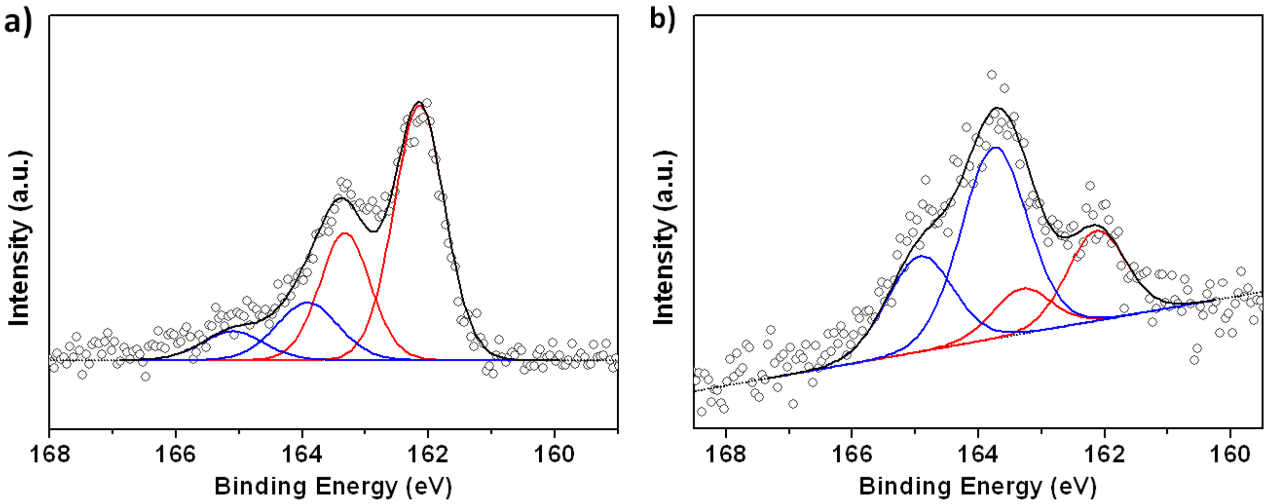

Supplementary Figure 10. S 2p XPS spectra for self-assembled films of (**a**) PPEs and (**b**) TTF-PPEs after oxidized with excess hexahydrate perchlorate iron.

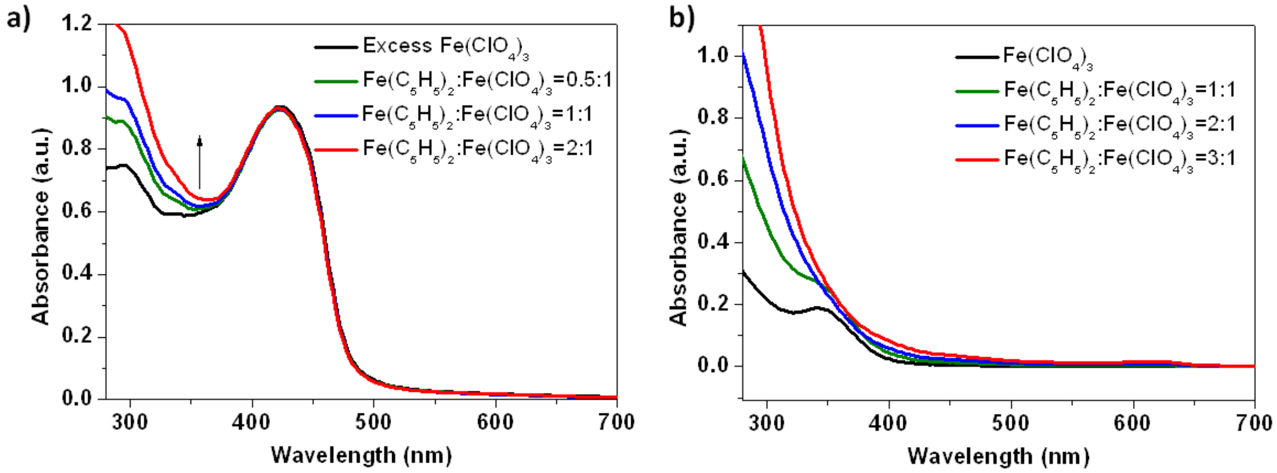

Supplementary Figure 11. (**a**) The change of UV-*vis* absorption spectra of TTF-PPEs (in dry THF, 298 K) after adding excess iron perchlorate hexahydrate $Fe(ClO_4)_3{\cdot}6H_2O$ and then adding different equivalents of reducing agent ferrocene $Fe(C_5H_5)_2$. No clue of reduction is recorded. (**b**) Control experiments: UV-*vis* absorption spectra of $Fe(ClO_4)_3$ solution (10^{-4} M in dry THF, 298K, black line) and then adding different equivalents of reducing agent ferrocene $Fe(C_5H_5)_2$ (10^{-4} M in dry THF, 298K, colored lines).

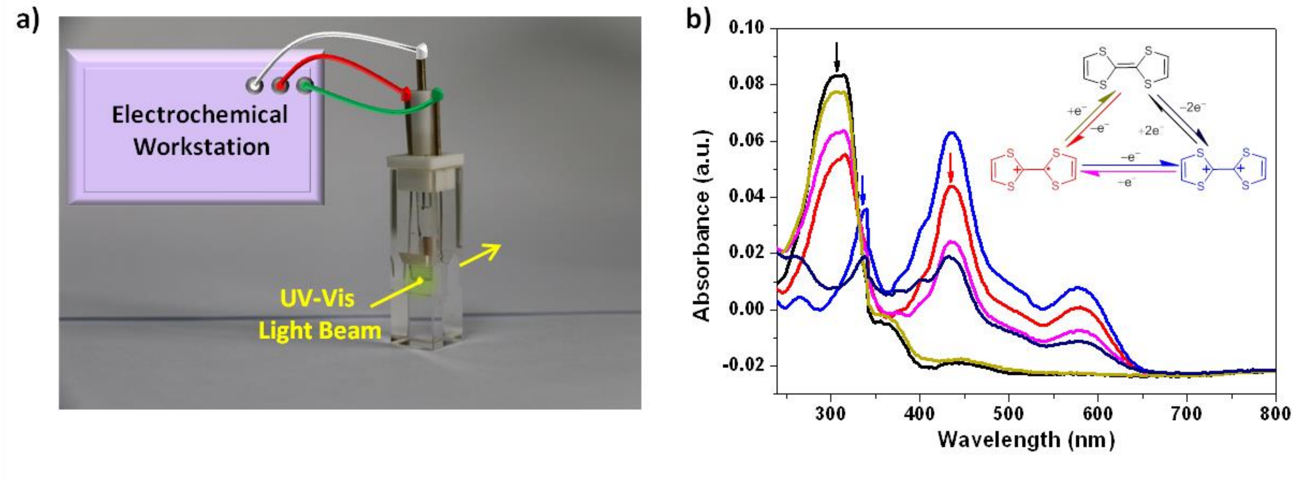

Supplementary Figure 12. (**a**) Test equipment chart for spectroelectrochemical method and in order to validate the feasibility of this method, the well-studied tetrathiafulvalene (TTF) is used for pretest. (**b**) Absorption spectra of TTF dilute solution in dry CH_3CN (black), after electrochemical oxidation at +0.3 V (*vs* Ag/AgCl) for 60 s (red, $TTF^{\bullet+}$), after electrochemical oxidation at +0.8 V (*vs* Ag/AgCl) for 240 s (blue, TTF^{2+}), after electrochemical reduction at -0.1 V (*vs* Ag/AgCl) for 600 s (magenta, $TTF^{\bullet+}$), after electrochemical reduction at -0.1 V (*vs* Ag/AgCl) for 600 s (dark yellow, TTF), and after electrochemical oxidation at +0.8 V (*vs* Ag/AgCl) for 600 s (navy, TTF^{2+}). The oxidation-reduction process can be reproduced at least two cycles[3].

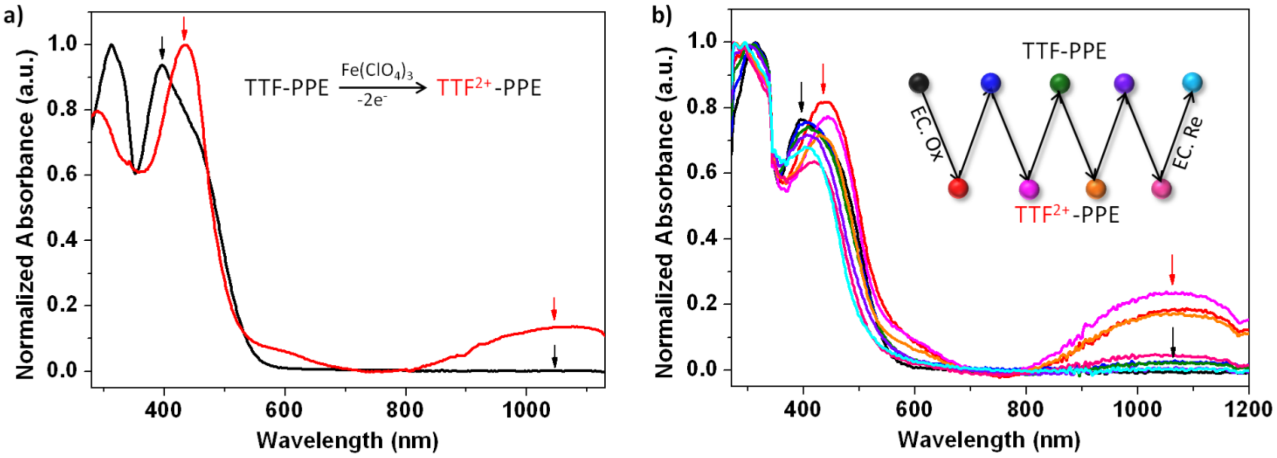

Supplementary Figure 13. (**a**) UV-*vis* absorption spectra changes (in dry THF, 298 K) upon successively adding oxidant $Fe(ClO_4)_3 \cdot 6H_2O$ in solution of TTF-PPE. The absoption of the initial state TTF-PPE (black) is at 396 nm, and after adding $Fe(ClO_4)_3 \cdot 6H_2O$, the new absorption of 429 nm and a broad absorption around 1040 nm appear, indicating the final dication state TTF^{2+}-PPE (red)[4]. (**b**) UV-*vis* absorption spectra changes (in dry THF, 298K) of TTF-PPE upon successive electrochemical oxidation and reduction. The black line refers to the initial state (400 nm), and after electrochemical oxidation at +2.0 V (*vs* Ag/AgCl) for 10 min, it transforms to the dication state TTF^{2+}-PPE (red, ~ 437 nm peak and around 1050 nm broad absorption), and after electrochemical reduction at -0.2 V (vs Ag/AgCl) for 30 min, it changes back to the neutral state (blue). Generally the electrochemical oxidation and reduction process can be repeated for 3-4 cycles. Then an apparent degradation of the system happens.

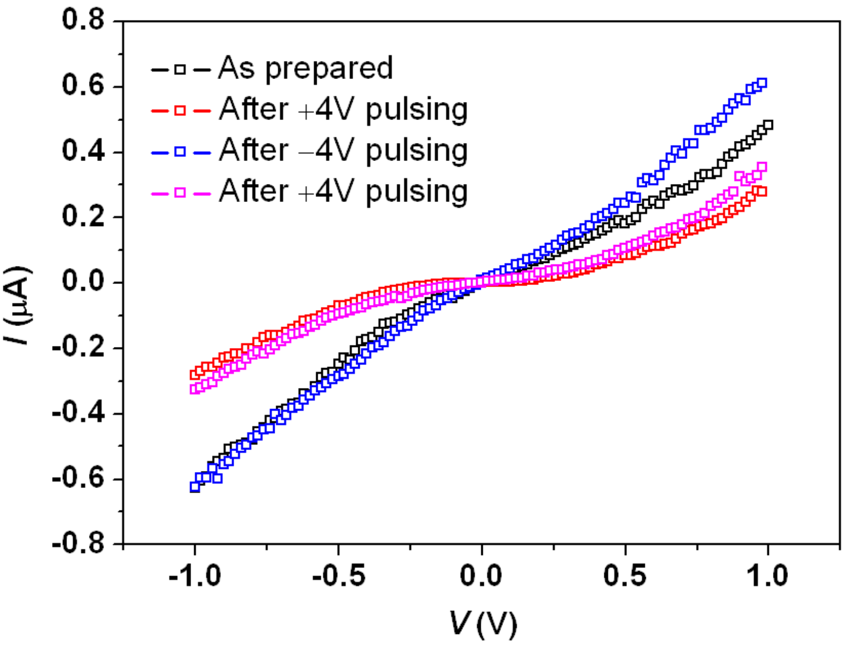

Supplementary Figure 14. *I*-*V* characteristics for voltage-driven reversible conductance switching of rGO/TTF-PPE CPs/Au junctions with two cycles of oxidation-reduction process. The voltages are swept from -1.0 to 1.0 V after applying pulsed voltges of +4 V and -4 V for 30 s, respectively.

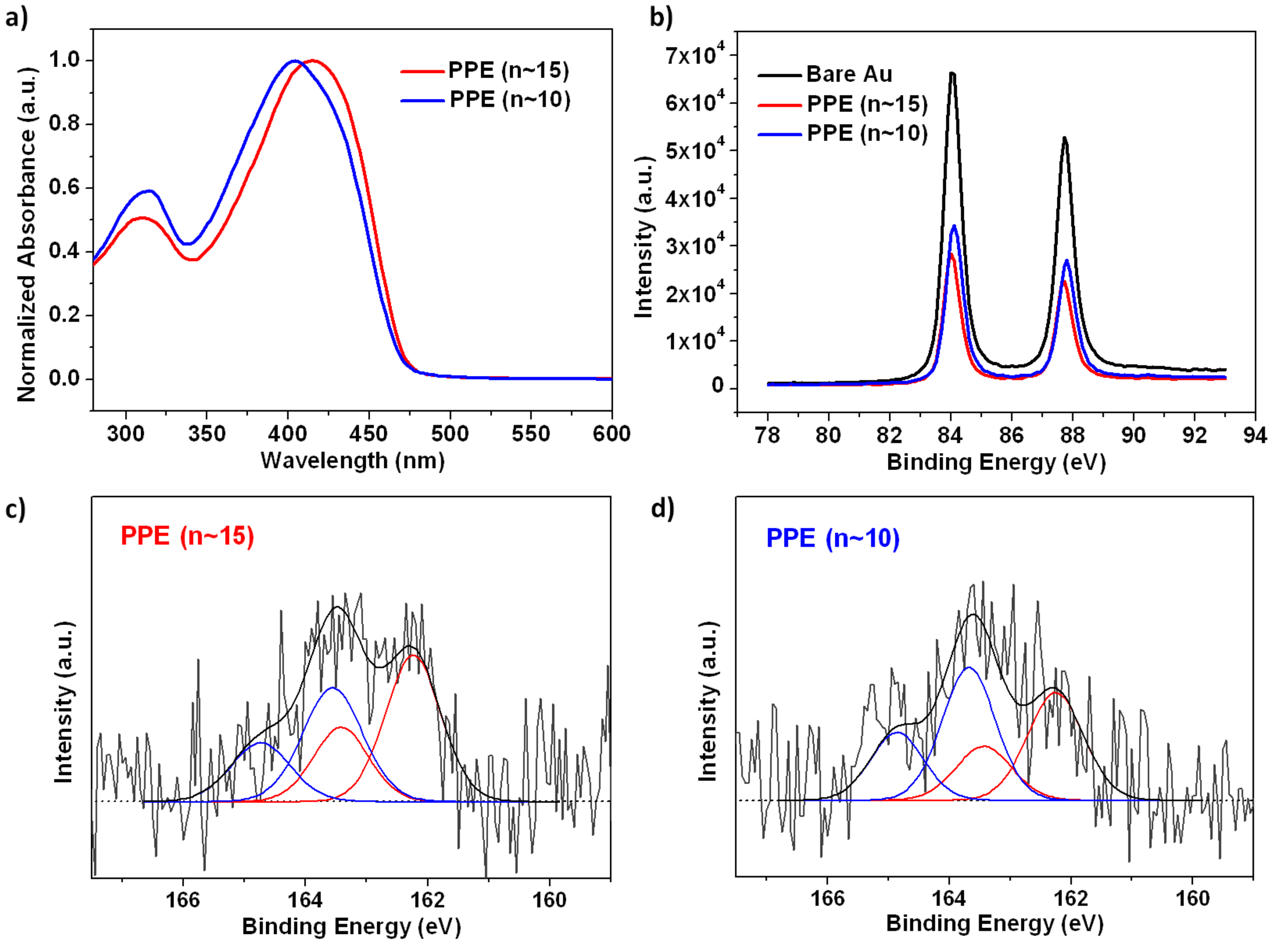

Supplementary Figure 15. Characterization of PPEs with low degree of polymerization (n ~ 15, red; n ~ 10, blue) in solution and SAMs on gold surface. (**a**) UV-*vis* absorption spectra of shorter PPEs. The red shift of main maximun absorption of PPE (n ~ 15) is due to an increased conjugation length compared with PPE (n ~ 10). (**b**) XPS spectra of Au 4f region for bare gold and with self-assembled PPEs (PPE (n ~ 15) thickness: 4.2 ± 0.2 nm; PPE (n ~ 10) thickness: 3.1 ± 0.2 nm). S 2p XPS spectra for self-assembled monolayers of (**c**) PPE (n ~ 15) and (**d**) PPE (n ~ 10) on Au substrate. The relative intensity of peaks for S-Au and S-R exhibit a ratio of about 1 : 1 and the thickness of shorter PPEs (PPE (n ~ 15): 4.2 ± 0.2 nm, PPE (n ~ 10): 3.1 ± 0.2 nm) is apparently larger than that of long PPEs (n ~ 100, thickness: 2.0 ± 0.2 nm), indicating that the two shorter PPEs are more inclined to stand up with a certain angle rather than totally lie flat on the Au surface.

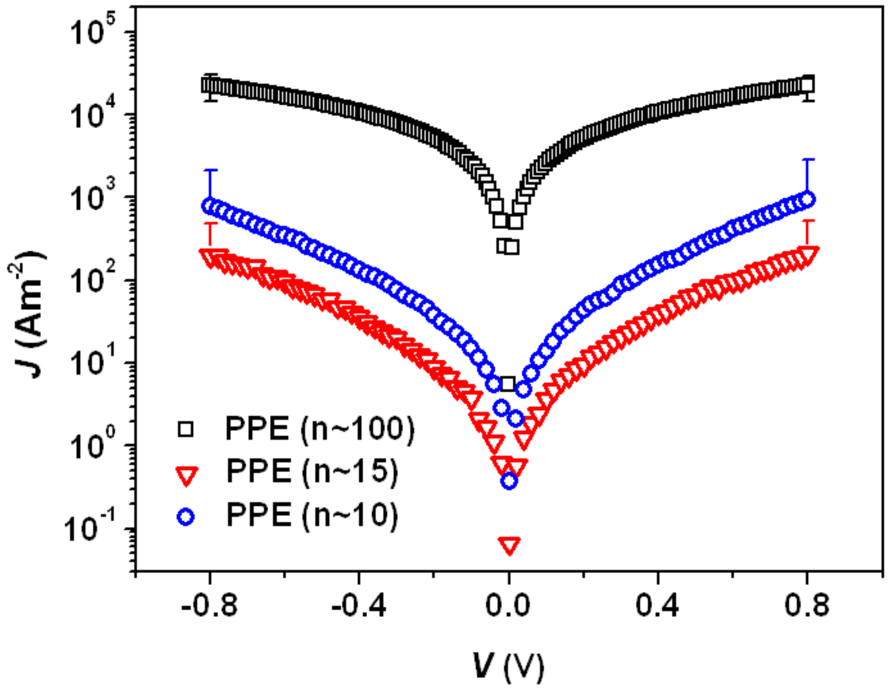

Supplementary Figure 16. *J-V* characteristics of junctions based on PPEs with different polymerization degree (DP): PPE (n ~ 100, black square), PPE (n ~ 15, red triangle) and PPE (n ~ 10, blue circle). The plots are generated from average values obtained from least 20 junctions of the same batch.

Supplementary References:

1. Lu, Q., Liu, K., Zhang, H., Du, Z., Wang, X. & Wang, F. Junctions based on oligo(p-phenylene ethynylene)s. *ACS Nano* **3**, 3861-3868 (2009).

2. Bain, C. D. & Whitesides, G. M. Attenuation lengths of photoelectrons in hydrocarbon films. *J. Phys. Chem.* **93**, 1670-1673 (1989).

3. Zhou, Y., Wu, H., Qu, L., Zhang, D. & Zhu, D. A new redox-resettable molecule-based half-adder with tetrathiafulvalene. *J. Phys. Chem. B* **110**, 15676-15679 (2006).

4. Halpin, Y. *et al*. Electrochemistry and time dependent DFT study of a (vinylenedithio)-TTF derivative in different oxidation states. *Electrochimica Acta* **100**, 188-196 (2013).
